# Breast-contour preserving procedures for early-stage breast cancer: a population-based study of the trends, variation in practice and predictive characteristics in Denmark and the Netherlands

**DOI:** 10.1007/s10549-020-05725-z

**Published:** 2020-06-10

**Authors:** E. Heeg, M. B. Jensen, M. A. M. Mureau, B. Ejlertsen, R. A. E. M. Tollenaar, P. M. Christiansen, M. T. F. D. Vrancken Peeters

**Affiliations:** 1 Dutch Institute for Clinical Auditing, Leiden, The Netherlands; 2 Department of Surgery, Leiden University Medical Centre, Albinusdreef 2, 2333 ZA Leiden, The Netherlands; 3 Danish Breast Cancer Cooperative Group, Copenhagen University Hospital, Rigshospitalet, Copenhagen, Denmark; 4 Department of Plastic and Reconstructive Surgery, Erasmus MC Cancer Institute, University Medical Centre Rotterdam, Rotterdam, The Netherlands; 5 Danish Breast Cancer Cooperative Group, Department of Oncology, Copenhagen University Hospital, Rigshospitalet, Copenhagen, Denmark; 6 Department of Plastic and Breast Surgery, Aarhus University Hospital, Aarhus, Denmark; 7 Department of Surgery, Netherlands Cancer Institute, Amsterdam, The Netherlands

**Keywords:** Breast cancer, Breast-contour preservation, Population-based, Neoadjuvant chemotherapy, Immediate breast reconstruction

## Abstract

**Purpose:**

Breast-contour preservation (BCP) is possible for most women treated for early-stage breast cancer. BCP can be defined as primary breast-conserving treatment (BCT), neoadjuvant chemotherapy (NAC) followed by BCT and immediate postmastectomy breast reconstruction (IBR). This study provides insight in current BCP strategies in Denmark and the Netherlands and aims to identify opportunities for improvement within both countries.

**Methods:**

A total of 92,881 patients with early-stage breast cancer who were operated in Denmark and the Netherlands between 2012 and 2017 were selected from the Danish Breast Cancer Group and the Dutch National Breast Cancer Audit databases. BCP procedures and predictive factors were analyzed within and between both countries.

**Results:**

BCP was achieved in 76.7% (*n* = 16,355) of the Danish and in 74.5% (*n* = 53,328) of the Dutch patients. While BCP rate did not change significantly over time in Denmark (*p* = 0.250), a significant increase in BCP rate from 69.5% in 2012 to 78.5% in 2017 (*p* < 0.001) was observed in the Netherlands. In both countries, variation in BCP rates between hospitals decreased over time. NAC followed by BCT and postmastectomy IBR was substantially more often used in the Netherlands compared to Denmark, specifically in patients younger than 50 years.

**Conclusions:**

In more than 75% of all Danish and Dutch patients, surgically treated for early-stage breast cancer, the breast-contour was preserved. The different use of BCP strategies within Denmark and the Netherlands and the differences observed between hospitals in both countries emphasize the need for more (inter)national consensus on treatment modalities.

## Introduction

Since several landmark studies in the 1980s confirmed comparable survival outcomes for early-stage breast cancer after breast-conserving treatment (BCT) and mastectomy [1–3], BCT has become the preferred standard of care. BCT is defined as breast-conserving surgery (BCS) followed by radiation therapy. Besides the surgical shift towards BCS, attention for outcomes such as the patients’ quality of life has increased in the last decade.

Loss of the breast mound may lead to a decreased self-image and quality of life [4]. Nowadays, preservation of the breast mound is possible for most early-stage breast cancer patients. This has been achieved by primary BCS for smaller tumors, and by the introduction of oncoplastic surgery techniques for large tumors [5]. Furthermore, neoadjuvant chemotherapy (NAC) is increasingly being used to downsize the tumor allowing for BCT [6]. In addition to this, immediate breast reconstruction (IBR) leads to restoration of the breast mound if mastectomy is indicated. Furthermore, IBR compared to delayed breast reconstruction reduces additional operations and hospitalizations [7], leading to reduced patient burden and healthcare costs [8].

National registries have demonstrated that overall, the proportion of breast cancer patients undergoing BCT (either primary or after NAC) or mastectomy followed by IBR, is currently over 70% in the USA and some European countries [9–13]. It is also known that there is a large variation between hospitals in the use of different treatment modalities [14, 15].

A previous study reported on breast-contour preservation (BCP) as a new comprehensive parameter for evaluating breast cancer treatment on a national level [16]. This new parameter aims to reflect the combined efforts of nonsurgical and surgical treatments to achieve preservation of the breast mound and is defined as either primary BCS, BCS after NAC or mastectomy followed by IBR. Because not all patients are primarily eligible for BCS, increasing the rate of BCP using either NAC or mastectomy followed by IBR is therefore of major importance. Despite that European, Danish and Dutch guidelines empathize the importance of BCP by highlighting the separate treatment strategies leading to BCP [17–19], previous literature focusing on all different strategies is sparse and nonexisting when comparing countries within Europe.

An international evaluation of BCP rates could provide valuable insight into daily practice and could identify possibilities to improve BCP strategies. The Netherlands and Denmark are both high-income countries and have well-maintained population-based breast cancer registries with similar medical care systems that provide equal access to healthcare for all patients, making them suitable for evaluation. The aims of the current study were to investigate the prevalence of BCP among women with early-stage breast cancer in Denmark and the Netherlands and to identify opportunities for improvement. This information can be used to increase the use of BCP procedures and reduce variation between hospitals in BCP rate in patients with early-stage breast cancer.

## Methods

### Data sources

Anonymized patient data regarding the demographic, clinicopathological, and treatment characteristics was obtained from the Danish Breast Cancer Group (DBCG) and the National Breast Cancer Organization Netherlands (NABON) Breast Cancer Audit (NBCA) from Denmark and the Netherlands, respectively. The scientific committee of the NBCA and the DBCG Board, and the Danish Clinical Quality Program– National Clinical Registries (RKKP) approved this study.

The DBCG was established in 1977 and prospectively collects data on patient-, tumor, and treatment-related characteristics, and follow-up of all female patients diagnosed with primary breast cancer in Denmark [20]. The NBCA was established in 2011 and prospectively collects data on patient-, tumor-, and treatment-related characteristics of all surgically treated patients diagnosed with primary invasive breast cancer or ductal carcinoma in situ (DCIS) in the Netherlands [9]. A more detailed description of the organization and data collection of both the DBCG and NBCA was published previously [9, 21].

### Study population

All female patients with primary invasive early-stage breast cancer who were operated between January 1st, 2012 and December 31st, 2017 and were registered in the DBCG or NBCA database were selected for this study. Early-stage breast cancer was defined as T1-2 N0-1 without distant metastasis. Patients diagnosed with locally advanced breast cancer were excluded. Patients diagnosed with only DCIS were not included as these patients are not registered in the database for the clinical quality program in Denmark, and therefore completeness of data is uncertain.

### Definitions and outcomes

In both countries, rarely reported histological subtypes such as mucinous, medullary, papillary, and tubular subtype were categorized as ‘other.' In Denmark, differentiation grade was determined for ductal and lobular breast cancer, but not for other subtypes according to the modified version of the Bloom-Richardson scoring system by Ellis et al*.* [22]. In the Netherlands, differentiation grade was categorized for all histological subtypes according to the modified version by Lakhani et al*.* [23]. In both registers, tumor size and lymph node status was categorized according to the 7th edition of the American Joint Committee on Cancer’s Cancer Staging Manuel [24]. For the current study, breast cancer specimens with ≥ 10% positively stained cells for estrogen receptor (ER) by immunohistochemistry were considered positive. Progesterone status is not registered in the DBCG database for the clinical quality program in Denmark and could therefore not be included in the current study. Human epidermal growth factor receptor 2 (HER2) expression was tested using an immunohistochemistry test or gene amplification in a fluorescence in situ hybridization test according to standard criteria [25].

Surgical treatment was categorized as BCS or mastectomy at definitive treatment. The primary outcome of this study was preservation of the breast contour. A definition of BCP was met if the patient underwent one of the following treatments: (1) primary BCS, (2) NAC followed by BCS, or (3) mastectomy (either primary or after NAC) followed by IBR. IBR was defined as breast reconstruction at the same procedure as mastectomy. Patients were categorized as not having received BCP if they had undergone mastectomy without IBR. While in the DBCG database, surgical procedures up to 1 month following primary surgery are included, no time limit exists for inclusion of secondary procedures in the NBCA database for the primary breast tumor.

Hospital surgical volume was defined as the average number of included patients operated per hospital per year and was categorized in low (< 150 patients), intermediate (150–299 patients), and high (≥ 300 patients) volume hospital. The average number of patients was for hospitals that were not active the whole period, only accounted for the years the hospitals actually treated patients.

### Statistical analysis

Analyses were stratified into two patient populations: (1) patients registered in the DBCG database and (2) patients registered in the NBCA database. Missing characteristics were categorized as a separate characteristic. Patient-, tumor-, and hospital-related characteristics were compared between patients who underwent mastectomy alone and those who underwent a BCP procedure, using *χ*^2^-tests for categorical variables. Patients with unknown characteristics were included in the descriptive statistics. Descriptive statistics were used to report on the overall BCP rate in both populations. To describe the BCP rate between hospitals and over time, hospital mean and 95% control limits (CLs) are presented in three funnel plots for year of operation 2013, 2015, and 2017 [26, 27]. Univariable and multivariable logistic regression analyses were used to estimate the odds ratio (OR) with 95% confidence intervals (CIs) for BCP, applying the Wald test for statistics significance. Patients with unknown characteristics were not included in univariable and multivariable analyses. All tests were two-sided and a *p* value of < 0.05 was considered statistically significant. Analyses were performed using SPSS ® (version 24, IBM, Armonk, New York, USA).

## Data availability

Data can be made available upon reasonable request to the NBCA and the DBCG Board, and the Danish Clinical Registries.

## Results

In total, 92,881 patients met the inclusion criteria, of whom 21,288 (22.9%) had been registered in Denmark and 71,593 (77.1%) in the Netherlands. The mean age (standard deviation) at diagnosis was 61.7 (12.5) years for patients in Denmark and 61.1 (12.3) years for patients in the Netherlands. In both countries, most of the patients were diagnosed with stage T1 (≤ 20 mm) breast cancer without lymph node involvement and with a ductal subtype which was estrogen positive and HER2 negative (Table 1).

**Table 1:** Baseline characteristics of patients diagnosed with early-stage breast cancer in Denmark and the Netherlands who underwent a breast-contour preserving procedure or mastectomy alone between 2012 and 2017

	Denmark (*n* = 21,288)				Netherlands (*n* = 71,593)			
		Breast-contour preservation				Breast-contour preservation		
	*N* (col %)	No (row %)	Yes (row %)	*p* value	*N* (col %)	No (row %)	Yes (row %)	*p* value
Number of patients	21,288	23.3	76.7		71,593	25.5	74.5	
Year of operation								
2012	3455 (16.2)	24.2	75.8	0.25	11,412 (15.9)	30.5	69.5	< 0.001
2013	3568 (16.8)	24.1	75.9		11,586 (16.2)	29.0	71.0	
2014	3617 (17.0)	22.3	77.7		11,985 (16.7)	27.0	73.0	
2015	3552 (16.7)	22.4	77.6		11,889 (16.6)	24.3	75.7	
2016	3529 (16.6)	23.4	76.6		12,313 (17.2)	21.4	78.6	
2017	3567 (16.8)	23.2	76.8		12,408 (17.3)	21.5	78.5	
Age (years)								
< 40	838 (3.9)	30.8	69.2	< 0.001	2850 (4.0)	22.5	77.5	< 0.001
40–49	2857 (13.4)	25.3	74.7		9818 (13.7)	23.0	77.0	
50–59	5087 (23.9)	17.1	82.9		18,870 (26.4)	18.6	81.4	
60–69	7023 (33.0)	16.8	83.2		21,189 (29.6)	22.0	78.0	
70–79	3827 (18.0)	30.7	69.3		13,943 (19.5)	30.6	69.4	
≥ 80	1656 (7.8)	45.2	54.8		4923 (6.9)	59.4	40.6	
Histological subtype								
Ductal	17,012 (79.9)	22.1	77.9	< 0.001	58,362 (81.5)	23.9	76.1	< 0.001
Lobular	2220 (10.4)	31.7	68.3		7555 (10.6)	36.4	63.6	
Other	2046 (9.6)	23.4	76.6		5242 (7.3)	28.3	71.7	
Unknown	10 (0.0)	60.0	40.0		434 (0.6)	23.0	77.0	
Differentiation grade								
I	5355 (25.2)	16.3	83.7	< 0.001	18,077 (25.2)	18.6	81.4	< 0.001
II	9203 (43.2)	24.7	75.3		32,243 (45.0)	26.6	73.4	
III	4407 (20.7)	27.7	72.3		16,663 (23.3)	29.7	70.3	
Not determined	2046 (9.6)	23.4	76.6		–	–	–	
Unknown	277 (1.3)	36.1	63.9		4610 (6.4)	30.0	70.0	
Estrogen receptor								
< 10%	3036 (14.3)	28.3	71.7	< 0.001	10,868 (15.2)	29.6	70.4	< 0.001
≥ 10%	18,198 (85.5)	22.4	77.6		59,114 (82.6)	24.8	75.2	
Unknown	54 (0.3)	31.5	68.5		1611 (2.3)	24.6	75.4	
HER2 status								
Negative	18,433 (86.6)	21.8	78.2	< 0.001	62,047 (86.7)	24.9	75.1	< 0.001
Positive	2622 (12.3)	32.8	67.2		8219 (11.5)	30.5	69.5	
Unknown	233 (1.1)	33.5	66.5		1327 (1.9)	23.7	76.3	
T-stage								
pT1	14,954 (70.2)	14.5	85.5	< 0.001	51,155 (71.5)	18.8	81.2	< 0.001
pT2	6225 (29.2)	43.4	56.6		20,360 (28.4)	42.3	57.7	
Unknown	109 (0.5)	73.4	26.6		78 (0.1)	42.3	57.7	
Lymph node status								
pN0	14,312 (67.2)	17.7	82.3	< 0.001	53,385 (74.6)	21.7	78.3	< 0.001
pN1	6262 (29.4)	35.1	64.9		18,089 (25.3)	36.5	63.5	
Unknown	714 (3.4)	31.5	68.5		119 (0.2)	51.3	48.7	
Hospital volume								
Low	703 (3.3)	17.8	82.2	< 0.001	28,608 (40.0)	30.2	69.8	< 0.001
Intermediate	8689 (40.8)	27.1	72.9		34,909 (48.8)	22.8	77.2	
High	11,896 (55.9)	20.8	79.2		8076 (11.3)	20.8	79.2	

*HER2* human epidermal growth factor receptor 2

In Denmark (*n* = 12 hospitals), there were 1 low-volume, 7 intermediate-volume, and 4 high-volume hospitals, whereas in the Netherlands (*n* = 82 hospitals), there were 50 low-volume, 28 intermediate-volume and 4 high-volume hospitals.

**Fig. 1:**
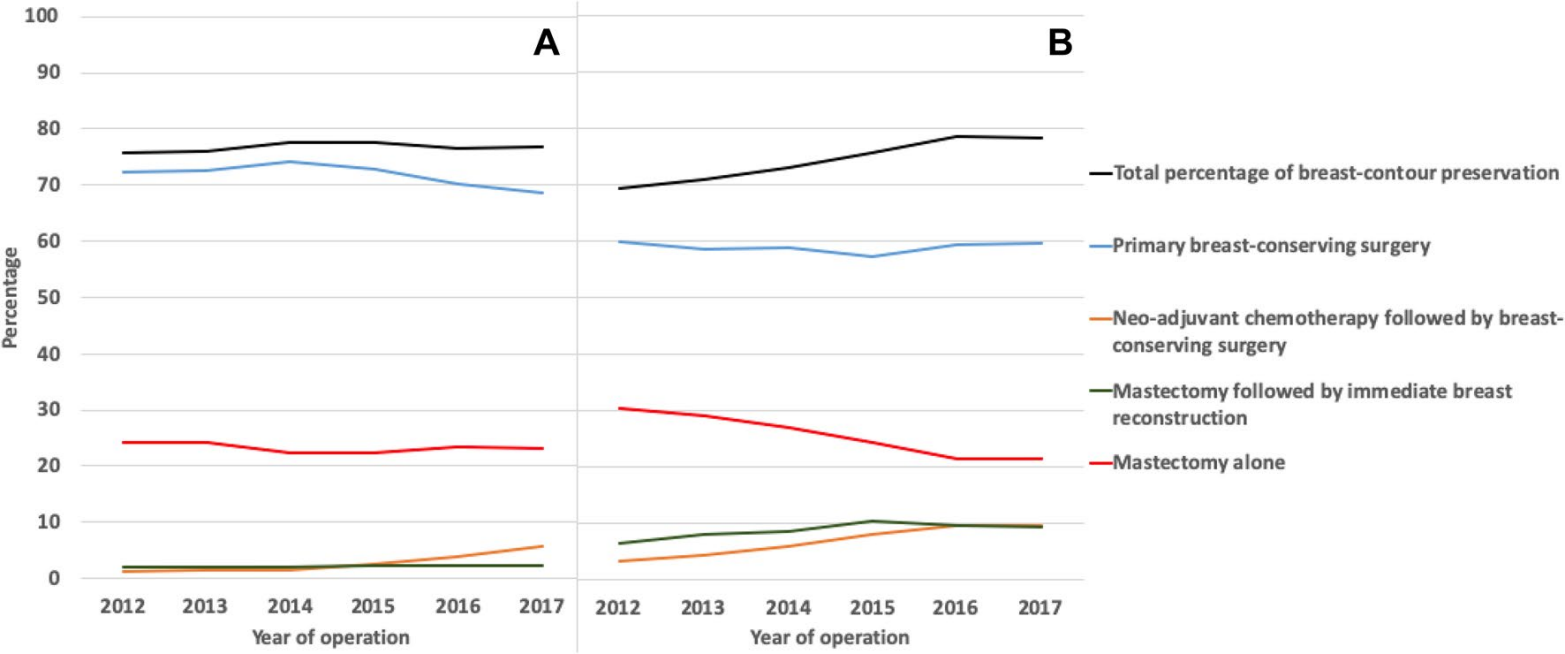
Breast cancer treatment strategies of patients diagnosed with early-stage breast cancer in **a** Denmark and **b** the Netherlands between 2012 and 2017

Between 2012 and 2017, 16,355 (76.7%) patients from Denmark and 53,328 (74.5%) patients from the Netherlands underwent BCP (Fig. 1). While the overall BCP rate was stable over time in Denmark (75.8% to 76.8%, *p* = 0.250), BCP rate increased significantly from 69.5% in 2012 to 78.5% in 2017 in the Netherlands (*p* < 0.001).

The BCP strategies changed significantly over time within both countries (*p* < 0.001). While the primary BCS rate decreased from 72.4% in 2012 to 68.7% in 2017 in Denmark, primary BCS rate only slightly decreased from 59.9 to 59.6% in the Netherlands (Fig. 1). The NAC followed by BCS rate increased from 1.3 to 5.7% in Denmark between 2012 and 2017 and from 3.1 to 9.6% in the Netherlands. The mastectomy followed by IBR rate slightly increased from 2.0% in 2012 to 2.4% in 2017 in Denmark and increased from 6.5 to 9.2% in the Netherlands (Fig. 1). The average mastectomy followed by IBR rate was different between both countries in both the lymph node positive patient group (1.5% in Denmark vs 9.3% in the Netherlands,) as in the lymph node negative patient group (2.4% vs 8.5%, respectively). In both countries, the majority of IBRs were implant- or tissue expander (TE)-based reconstructions (94.0 vs. 89.4%).

Baseline characteristics associated with BCP within both countries are listed in Table 2. While the year of diagnosis was not associated with BCP in Denmark, patients operated in more recent years compared to year 2012 were more likely to undergo a BCP procedure in the Netherlands, with increasing ORs (Table 2).

**Table 2:** Univariable and multivariable analyses of characteristics associated with breast-contour preservation within Denmark and the Netherlands

	Denmark (*n* = 20,096)			Netherlands (*n* = 64,594)		
	OR (95% CI) univariable	OR (95% CI) multivariable	*p* value	OR (95% CI) univariable	OR (95% CI) multivariable	*p* value
Year of diagnosis			0.07			< 0.001
2012	1.00 (reference)	1.00 (reference)		1.00 (reference)	1.00 (reference)	
2013	0.99 (0.88–1.11)	0.94 (0.83–1.06)		1.08 (1.01–1.14)	1.05 (0.98–1.12)	
2014	1.08 (0.96–1.21)	1.09 (0.96–1.23)		1.21 (1.14–1.28)	**1.20 (1.13–1.28)**	
2015	1.10 (0.98–1.24)	1.12 (0.98–1.27)		1.35(1.27–1.43)	**1.34 (1.25–1.43)**	
2016	1.02 (0.91–1.15)	1.07 (0.95–1.22)		1.57 (1.47–1.67)	**1.63 (1.52–1.74)**	
2017	1.02 (0.91–1.14)	1.05 (0.93–1.20)		1.56(1.47–1.66)	**1.59 (1.49–1.70)**	
Age (years)			< 0.001			< 0.001
< 40	0.45 (0.38–0.53)	**0.64 (0.53–0.77)**		0.97 (0.88–1.08)	**1.20 (1.08–1.34)**	
40–49	0.61 (0.55–0.68)	**0.84 (0.74–0.94)**		0.96 (0.90–1.02)	**1.20 (1.12–1.28)**	
50–59	0.99 (0.89–1.09)	**1.13 (1.02–1.26)**		1.23 (1.17–1.29)	**1.31 (1.25–1.39)**	
60–69	1.00 (reference)	1.00 (reference)		1.00 (reference)	1.00 (reference)	
70–80	0.45 (0.41–0.49)	**0.55 (0.49–0.61)**		0.63 (0.60–0.66)	**0.62 (0.59–0.66)**	
≥ 80	0.23 (0.20–0.26)	**0.30 (0.26–0.34)**		0.19 (0.18–0.20)	**0.24 (0.22–0.26)**	
Histological subtype			< 0.001			< 0.001
Ductal	1.00 (reference)	1.00 (reference)		1.00 (reference)	1.00 (reference)	
Lobular	0.61 (0.56–0.68)	**0.62 (0.56–0.70)**		0.54 (0.51–0.57)	**0.58 (0.55–0.62)**	
Other	0.96 (0.85–1.08)	0.98 (0.84–1.14)		0.79 (0.79–0.84)	**0.80 (0.74–0.86)**	
Differentiation grade			0.002			< 0.001
I	1.00 (reference)	1.00 (reference)		1.00 (reference)	1.00 (reference)	
II	0.67 (0.62–0.73)	**0.84 (0.77–0.93)**		0.63 (0.60–0.66)	**0.80 (0.84–0.84)**	
III	0.57 (0.52–0.62)	0.92 (0.81–1.04)		0.54 (0.51–0.57)	**0.76 (0.81–0.81)**	
Estrogen receptor			0.392			< 0.001
< 10%	0.75 (0.69–0.82)	0.95 (0.85–1.07)		0.77 (0.74–0.81)	**0.89 (0.83–0.94)**	
≥ 10%	1.00 (reference)	1.00 (reference)		1.00 (reference)	1.00 (reference)	
HER2 status			< 0.001			< 0.001
Negative	1.00 (reference)	1.00 (reference)		1.00 (reference)	1.00 (reference)	
Positive	0.56 (0.51–0.62)	**0.58 (0.52–0.65)**		0.75 (0.71–0.79)	**0.73 (0.69–0.78)**	
T-stage			< 0.001			< 0.001
pT1	1.00 (reference)	1.00 (reference)		1.00 (reference)	1.00 (reference)	
pT2	0.21 (0.20–0.23)	**0.28 (0.26–0.30)**		0.30 (0.29–0.31)	**0.40 (0.38–0.41)**	
Lymph node status			< 0.001			< 0.001
pN0	1.00 (reference)	1.00 (reference)		1.00 (reference)	1.00 (reference)	
pN1	0.41 (0.38–0.44)	**0.51 (0.48–0.55)**		0.48 (0.46–0.50)	**0.53 (0.51–0.56)**	
Hospital volume			< 0.001			< 0.001
Low	1.73 (1.40–2.13)	**1.91 (1.52–2.40)**		0.67 (0.65–0.70)	**0.66 (0.64–0.69)**	
Intermediate	1.00 (reference)	1.00 (reference)		1.00 (reference)	1.00 (reference)	
High	1.45 (1.36–1.55)	**1.55 (1.44–1.67)**		1.13(1.06–1.20)	**1.09 (1.02–1.17)**	

Odds ratios presented in bold were statistically significant

*HER2* human epidermal growth factor receptor 2

The overall BCP rate was significantly different between age groups (Table 1). After adjusting for confounders, in both countries, patients between 50 and 59 years old were more likely to undergo BCP compared to patients of 60 to 69 years of age (OR 1.13, 95% CIs 1.02–1.26 and OR 1.31, 95% CIs 1.25–1.39, respectively). In the Netherlands, patients younger than 40 years and between 40 to 49 years of age were also more likely to undergo BCP compared to those who were 60 to 69 years old (OR 1.20, 95% CIs 1.08–1.34 and OR 1.20, 95% CIs 1.12–1.28, respectively). Whereas, in Denmark, patients younger than 40 years and between 40 and 49 years of age (OR 0.64, 95% CIs 0.53–0.77 and OR 0.84, 95% CIs 0.74–0.94, respectively) were less likely to undergo BCP compared to those who were 60 to 69 years old (Table 2).

Among other predictive characteristics, patients diagnosed with a T2 tumor (OR 0.28, 95% CIs 0.26–0.30 and OR 0.40, 95% CIs 0.38–0.41, respectively) compared to T1 tumor and lymph node involvement (OR 0.51, 95% CIs 0.48–0.55 and OR 0.53, 95% CIs 0.51–0.56, respectively) were less likely to undergo BCP within both Denmark and the Netherlands.

**Fig. 2:**
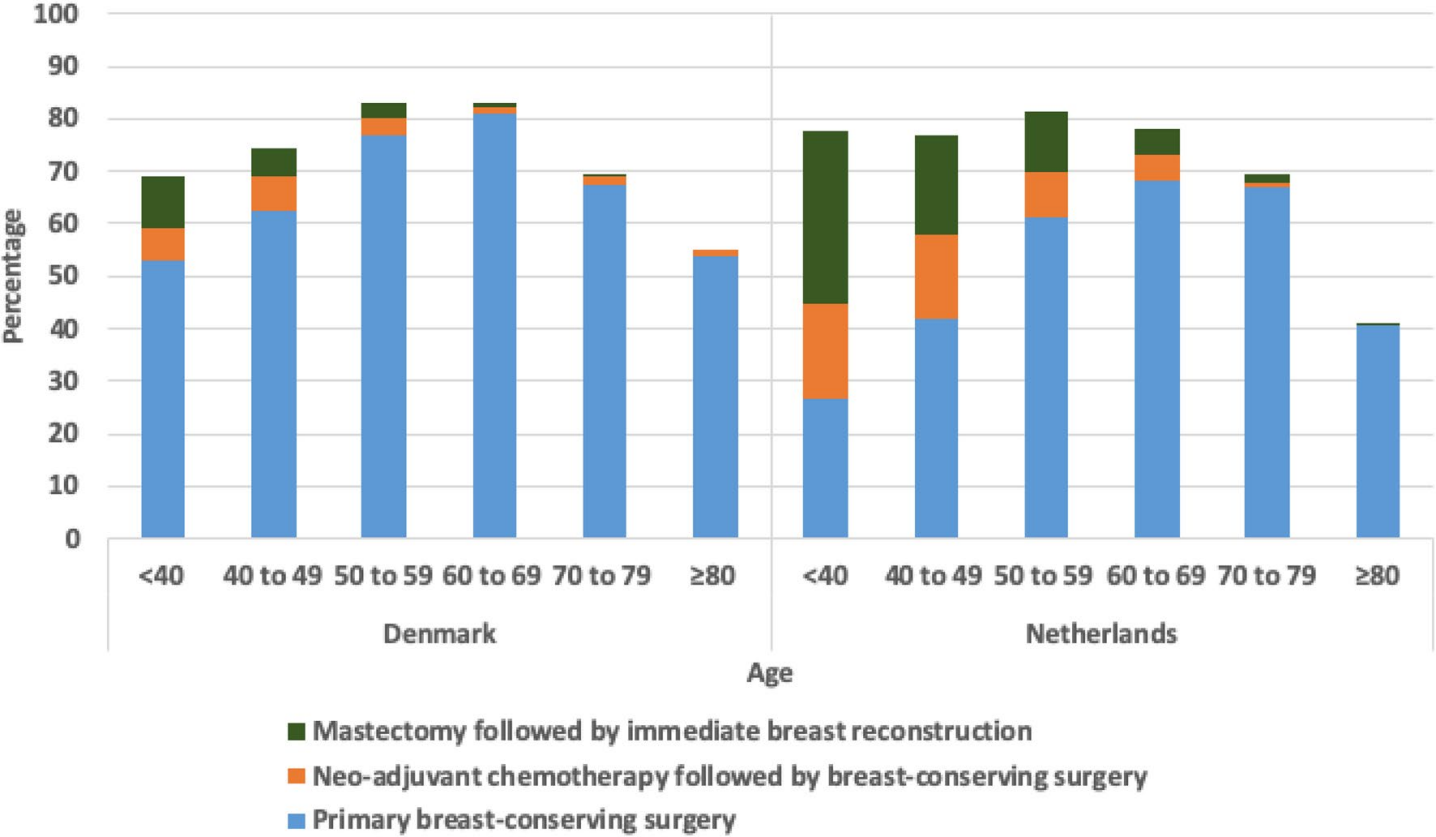
Breast-contour preservation strategies per age group for patients with early-stage breast cancer in Denmark and the Netherlands

In both Denmark and the Netherlands, NAC followed by BCS (6.1% and 18.2%, respectively) and mastectomy followed by IBR (10.1% and 32.9%, respectively) was most commonly performed in patients younger than 40 years (Fig. 2). Both treatment modalities were less commonly performed as age increased and were almost never performed in patients older than 80 years (Fig. 2).

**Fig. 3:**
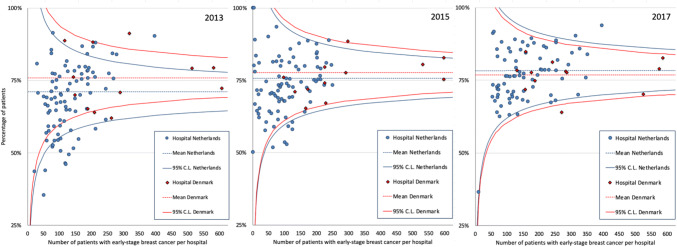
Funnel plots of breast-contour preservation for early-stage breast cancer in Denmark (red) and the Netherlands (blue) in 2013, 2015 and 2017, showing the hospital mean and 95% control limits. Note: the y-axis starts from 25%

Although in general the variation in BCP rates between hospitals was smaller in Denmark compared to the Netherlands, a decrease in variation in BCP rates was observed between hospitals within both Denmark and the Netherlands over time (Fig. 3).

In both countries, patients were more likely to undergo BCP if they underwent surgery at a high-volume hospital (OR 1.55, 95% CIs 1.44–1.67 and OR 1.09, 95% CIs 1.02–1.17, respectively) compared to an intermediate-volume hospital. In the Netherlands, patients treated at a low-volume hospital were less likely to preserve their breast contour compared to patients in an intermediate-volume hospital (OR 0.66, 95% CIs 0.64–0.69).

## Discussion

In this large population-based study of patients with early-stage breast cancer, insight into BCP strategies is provided and it is shown that BCP was achieved in more than 75% of patients in both Denmark and the Netherlands, albeit using different treatment strategies. While in Denmark BCP was predominantly achieved by using primary BCS, the use of NAC followed by BCS and mastectomy followed by IBR played a substantial role in the Netherlands, specifically in patients younger than 50 years. We observed a stable high overall BCP rate between 2012 and 2017 in Denmark and a significant increase over time in the Netherlands. Current findings demonstrated considerable variation in the use of BCP strategies between hospitals within both countries and between countries. These results therefore suggest that more (inter)national consensus on the indication for different breast cancer treatment modalities is warranted, specifically on indications for NAC and mastectomy followed by IBR.

Van Bommel et al*.* first described BCP as a new comprehensive parameter for evaluating quality of breast cancer treatment in patients with early-stage as well as more advanced stages of breast cancer [16]. They reported an increase in the overall BCP rate from 63% in 2011 to 71% in 2015 which was translated as a quality of care improvement [16]. This observed trend continued in the Netherlands up to 2017 as is shown in the current study, although the different strategies used in Denmark made an interesting perspective.

The current study observed differences in BCP strategies per age group and over time between Denmark and the Netherlands. The rate of patients undergoing NAC followed by BCS more than tripled in both countries over time. However, in Denmark, less patients younger than 50 years, underwent NAC followed by BCS (6.3 vs. 16.7%, respectively) or mastectomy followed by IBR (6.7 vs*.* 22.2%, respectively) compared to the Netherlands. In reviewing literature, several studies from around the world have reported an increasing use of NAC, specifically in patients younger than 70 years and in patients with more advanced tumors [6, 28, 29]. The difference in use of NAC followed by BCS between both countries may partly be explained by the moment of introducing NAC in the national guidelines. While in the Netherlands NAC was introduced as a downstaging procedure in the breast cancer guideline in 2012 [14, 30], Danish guidelines incorporated NAC as a downstaging procedure in the second half of 2016 and has been increasingly used thereafter [19].

Breast cancer with lymph node involvement requires radiotherapy, which limits the use of IBR as radiotherapy is frequently mentioned as a contraindication for implant-based IBR [31, 32]. Interestingly, different IBR rates among patients who underwent mastectomy were found between both countries, both in patients with a positive and negative lymph node. It is unlikely that the type of IBR technique explains the observed differences, since the majority of IBRs were implant- or TE based in both countries. This together with the relative low increase in IBR rates in Denmark suggests potential room for improvement in Denmark. Internationally, there has been an increasing use of IBR in most high-income countries in the last decade. The mean IBR rate of 25.5% among patients who underwent mastectomy in the Netherlands is within the range of other high-income countries, such as United Kingdom (up to 23% in 2016) [33], USA (up to 43% in 2014) [34], and Australia (up to 18% in 2013) [35]. Nonetheless, previous research has shown substantial variation in postmastectomy IBR rates between hospitals in the Netherlands and other countries, unexplained by patient and tumor characteristics [15, 36, 37]. The different use of IBR between hospitals emphasizes the need for more international consensus on the indications for IBR. Future cross-country studies could focus on hospital organizational factors as some of these are associated with the use of IBR [38, 39]. Unfortunately, these factors could not be accounted for in the current study.

Overall, a smaller proportion of patients underwent a mastectomy (with or without IBR) in Denmark compared to the Netherlands (25.5% vs. 34.2%, respectively). This finding suggest room for improvement in the Netherlands in performing more BCS instead of mastectomy, since previous studies showed comparable survival outcomes when comparing patients who underwent BCT and mastectomy [2, 40].

Different grading systems were used in both countries. Hereby, relatively more Dutch patients with an unknown differentiation grade were excluded from the logistic regression model compared to Danish patients. Nonetheless, the impact is most likely limited as subsequent analysis showed the same findings when including these patients (data not shown).

The current study highlights an interesting difference in hospital volume. While in Denmark only 3.3% of patients were operated at a low-volume hospital, this was 40% in the Netherlands. Despite that previous studies found minimal differences in survival between intermediate- and high-volume hospitals [41, 42], no literature exists on the relationship between hospital volume and ‘soft’ outcomes such as BCP. In the current study, a significant association between hospital volume and BCP rate was found. Although it is beyond the scope of the current study, current findings suggest that BCP might be increased in the Netherlands by centralizing breast cancer care. This hypothesis requires additional future analyses on the relationship between hospital volume and BCP.

A decrease of variation in BCP rates between hospitals was observed over time, specifically in the Netherlands. A potential contributor to this trend in the Netherlands might be the continuous feedback hospitals received on their BCP rate provided by the NBCA. Several other improvements in health care have been accomplished by monitoring the quality of cancer care and providing benchmark feedback to hospitals [43, 44].

The current study has several limitations. First, there might have been unaccounted confounders in the current analyses (e.g., comorbidities, social-economic status, smoking status). Unfortunately, these confounders are not registered in both databases. Second, there might be subtle differences in interpretation of definitions between those who register patients which might explain part of the treatment choices. Thirdly, only surgical procedures performed within 1 month after primary surgery were included in the DBCG database. Although most secondary surgery is performed within a short time period after primary surgery, secondary mastectomies or reconstructive efforts without oncologic purpose do occur after a longer time period, specifically in patients younger than 50 years with for instance a genetic predisposition. A previous study using the DBCG database reported a higher mastectomy rate after BCS in Denmark between 2008 and 2012 (data not shown) when including procedures up to 3 months after primary surgery [45]. Consensus regarding the inclusion period among national registries could improve future cross-country comparison. Lastly, the current study could unfortunately not account for whether patients in different hospitals had access to high skilled physicians who offered the entire field of breast reconstruction procedures. Strengths of the current study are the real-world population-based databases and high number of patients. To the best of the authors’ knowledge, there have been no previous cross-country population-based analyses, evaluating the comprehensive breast cancer treatment for early-stage breast cancer. Therefore, current findings can be used for comparison and benchmarking in future studies.

## Abbreviations

BCP: Breast-contour preservation
BCT: Breast-conserving treatment
NAC: Neoadjuvant chemotherapy
IBR: Immediate postmastectomy breast reconstruction
DBCG: Danish Breast Cancer Cooperative Group
NABON: National Breast Cancer Organization Netherlands
NBCA: NABON Breast Cancer Audit
DCIS: Ductal carcinoma in situ
ER: Estrogen receptor
HER2: Human epidermal growth factor receptor 2
OR: Odds ratio
CIs: Confidence intervals
